# Life-course inequalities in intrinsic capacity and healthy ageing, China

**DOI:** 10.2471/BLT.22.288888

**Published:** 2023-03-02

**Authors:** Yafei Si, Katja Hanewald, Shu Chen, Bingqin Li, Hazel Bateman, John R Beard

**Affiliations:** a School of Risk & Actuarial Studies, University of New South Wales, 223 Anzac Parade Kensington, Sydney NSW 2052, Australia.; b Social Policy Research Centre, University of New South Wales, Sydney, Australia.; c ARC Centre of Excellence in Population Ageing Research, University of New South Wales, Sydney, Australia.

## Abstract

**Objective:**

To investigate the contribution of early-life factors on intrinsic capacity of Chinese adults older than 45 years.

**Methods:**

We used data on 21 783 participants from waves 1 (2011) and 2 (2013) of the China Health and Retirement Longitudinal Study (CHARLS), who also participated in the 2014 CHARLS Life History Survey to calculate a previously validated measure of intrinsic capacity. We considered 11 early-life factors and investigated their direct association with participants’ intrinsic capacity later in life, as well as their indirect association through four current socioeconomic factors. We used multivariable linear regression and the decomposition of the concentration index to investigate the contribution of each determinant to intrinsic capacity inequalities.

**Findings:**

Participants with a favourable environment in early life (that is, parental education, childhood health and neighbourhood environment) had a significantly higher intrinsic capacity score in later life. For example, participants with a literate father recorded a 0.040 (95% confidence interval, CI: 0.020 to 0.051) higher intrinsic capacity score than those with an illiterate father. This inequality was greater for cognitive, sensory and psychological capacities than locomotion and vitality. Overall, early-life factors directly explained 13.92% (95% CI: 12.07 to 15.77) of intrinsic capacity inequalities, and a further 28.57% (95% CI: 28.19 to 28.95) of these inequalities through their influence on current socioeconomic inequalities.

**Conclusion:**

Unfavourable early-life factors appear to decrease late-life health status in China, particularly cognitive, sensory and psychological capacities, and these effects are exacerbated by cumulative socioeconomic inequalities over a person’s life course.

## Résumé

**Objectif:**

Examiner la contribution des facteurs en début de vie aux capacités intrinsèques des adultes de plus de 45 ans en Chine.

**Méthodes:**

Nous avons utilisé les données relatives à 21 783 participants des vagues 1 (2011) et 2 (2013) de l'Étude longitudinale sur la santé et la retraite en Chine (CHARLS); ils avaient également pris part à l'Enquête CHARLS sur le cycle de vie en 2014, qui visait à calculer une mesure préalablement validée des capacités intrinsèques. Nous avons ensuite retenu 11 facteurs en début de vie et analysé leurs liens directs avec les capacités intrinsèques des participants plus tard dans leur vie, mais aussi leurs liens indirects à travers quatre facteurs socioéconomiques actuels. Enfin, nous avons employé une régression linéaire multivariée et décomposé l'indice de concentration afin de définir la contribution de chaque déterminant dans les inégalités observées au niveau des capacités intrinsèques.

**Résultats:**

Les participants ayant débuté leur vie dans un environnement favorable (c'est-à-dire avec une éducation parentale, une bonne santé durant l'enfance et un bon cadre de vie) affichaient un score de capacité intrinsèque nettement plus élevé par la suite. Par exemple, les participants dont le père était instruit ont enregistré un score de capacité intrinsèque de 0,040 (intervalle de confiance de 95%, IC: 0,020 à 0,051) supérieur à ceux dont le père était analphabète. Cette inégalité s'est révélée davantage marquée pour les capacités cognitives, sensorielles et psychologiques que pour la locomotion et la vitalité. De manière générale, les facteurs en début de vie ont pu être directement associés à 13,92% (IC de 95%: 12,07 à 15,77) des inégalités en matière de capacités intrinsèques, tandis que 28,57% (IC de 95%: 28,19 à 28,95) de ces inégalités étaient liées à leur influence sur les inégalités socioéconomiques actuelles.

**Conclusion:**

Les facteurs défavorables en début de vie semblent entraîner une détérioration de l'état de santé ultérieur en Chine, en particulier des capacités cognitives, sensorielles et psychologiques. Ces effets sont exacerbés par les inégalités socioéconomiques qui viennent s'y ajouter au cours de la vie d'une personne.

## Resumen

**Objetivo:**

Investigar la contribución de los factores de los primeros años de vida en la capacidad intrínseca de los adultos chinos mayores de 45 años.

**Métodos:**

Se utilizaron datos de 21 783 participantes de las oleadas 1 (2011) y 2 (2013) del Estudio Longitudinal de Salud y Jubilación de China, que también participaron en la Encuesta de Antecedentes Vitales CHARLS de 2014 para calcular una medida previamente validada de la capacidad intrínseca. Se tuvieron en cuenta 11 factores de los primeros años de vida y se investigó su asociación directa con la capacidad intrínseca de los participantes más adelante en la vida, así como su asociación indirecta a través de cuatro factores socioeconómicos actuales. Se utilizó la regresión lineal multivariable y la descomposición del índice de concentración para investigar la contribución de cada determinante a las desigualdades en la capacidad intrínseca.

**Resultados:**

Los participantes con un entorno favorable en los primeros años de vida (es decir, educación de los padres, salud en la infancia y entorno del vecindario) tuvieron una puntuación de capacidad intrínseca significativamente más alta en etapas posteriores de la vida. Por ejemplo, los participantes con un padre alfabetizado registraron una puntuación de capacidad intrínseca 0,040 (intervalo de confianza del 95 %, IC: 0,020 a 0,051) mayor que los de un padre analfabeto. Esta desigualdad fue mayor para las capacidades cognitivas, sensoriales y psicológicas que para la locomoción y la vitalidad. En conjunto, los factores de los primeros años de vida explicaban directamente el 13,92 % (IC 95 %: 12,07 a 15,77) de las desigualdades de capacidad intrínseca y otro 28,57% (IC 95 %: 28,19 a 28,95) de estas desigualdades a través de su influencia en las desigualdades socioeconómicas actuales.

**Conclusión:**

En China, los factores desfavorables en los primeros años de vida parecen reducir el estado de salud al final de la vida, sobre todo las capacidades cognitivas, sensoriales y psicológicas, los que se ven exacerbados por las desigualdades socioeconómicas acumuladas a lo largo de la vida.

## ملخص

**الغرض:**

البحث في إسهام عوامل الحياة المبكرة في القدرة الذاتية للبالغين الصينيين الأكبر من 45 سنة.

**الطريقة:**

استخدمنا البيانات الخاصة بمشاركين يبلغ عددهم 21783 من الموجتين 1 (2011)، و2 (2013) في الدراسة الطولية عن الصحة والتقاعد في الصين، والذين شاركوا أيضًا في استبيان CHARLS عن تاريخ الحياة لعام 2014 لحساب قياس تم التحقق منه سابقًا للقدرة الذاتية. وضعنا في الاعتبار 11 عاملاً في الحياة المبكرة، وبحثنا في ارتباطها المباشر بالقدرة الذاتية للمشاركين في وقت لاحق من الحياة، إلى جانب ارتباطها غير المباشر من خلال أربعة عوامل اجتماعية واقتصادية حالية. استخدمنا التراجع الخطي متعدد المتغيرات، وتفكيك مؤشر التركيز للبحث في إسهام كل عامل محدد في جوانب عدم المساواة في القدرة الذاتية.

**النتائج:**

المشاركون الذين كانت لديهم بيئة مواتية في الحياة المبكرة (بمعنى تعليم الوالدين، والتمتع بالصحة في الطفولة، والبيئة المجاورة) حصلوا على درجة قدرة ذاتية أعلى بشكل ملحوظ في الحياة مع تقدم العمر. على سبيل المثال، حصل المشاركون الذين لديهم أب متعلم على درجة قدرة ذاتية أعلى بمقدار 0.040 (بفاصل ثقة مقداره %95: 0.020 إلى 0.051) عن أولئك الذين لديهم أب غير متعلم. هذا الجانب لعدم المساواة كان أكبر في القدرات المعرفية والحسية والنفسية منه في القدرات الحركية والحيوية. فُسرت عوامل الحياة المبكرة بشكل مباشر عمومًا %13.92 (بفاصل ثقة مقداره %95: 12.07 إلى 15.77) من جوانب عدم المساواة في القدرة الذاتية وتم تفسير نسبة أخرى تبلغ %28.57 (بفاصل ثقة مقداره %95: 28.19 إلى 28.95) من جوانب عدم المساواة هذه من خلال تأثيرها على جوانب عدم المساواة الاجتماعية والاقتصادية الحالية.

**الاستنتاج:**

يبدو أن عوامل الحياة المبكرة غير المواتية تسبب تراجع الحالة الصحية في الحياة مع تقدم العمر في الصين، وخاصةً القدرات المعرفية والحسية والنفسية، وتزداد هذه التأثيرات بجوانب عدم المساواة الاجتماعية والاقتصادية التراكمية على مسار حياة الشخص.

## 摘要

**目的:**

旨在调查早期生活因素对中国 45 岁以上成年人的内在能力的影响。

**方法:**

我们使用了来自中国健康与养老追踪调查中第一轮(2011 年)和第二轮(2013 年)的 21,783 名参与者的数据,这些参与者也参与了 2014 年 CHARLS 生命历程调查,以计算先前验证过的内在能力测量方法。我们考虑了 11 个早期生活因素,并调查了它们与参与者晚年内在能力的直接联系,以及它们与当前四个社会经济因素的间接联系。我们使用了多变量线性回归和集中指数分解法来研究每个决定因素对内在能力不平等的影响。

**结果:**

早期生活环境(即父母教育、童年健康和社区环境)良好的参与者,晚年的内在能力得分明显较高。例如,父亲有文化的参与者的内在能力得分比父亲为文盲的参与者要高 0.040(95% 置信区间,CI:0.020 至 0.051)。这种不平等在认知、感觉和心理能力方面比在运动和活力方面更为严重。总的来说,早期生活因素直接解释了 13.92%(95% CI:12.07 至 15.77)内在能力的不平等,并通过其对当前社会经济不平等的影响,进一步解释了 28.57%(95% CI:28.19 至 28.95)的不平等。

**结论:**

在中国,不利的早期生活因素会降低晚年的健康状况,尤其在认知、感觉和心理能力方面,而这些影响又因一个人生命历程中社会经济的不平等而变得更加严重。

## Резюме

**Цель:**

Изучить влияние факторов в раннем периоде жизни на индивидуальную жизнеспособность взрослых китайцев старше 45 лет.

**Методы:**

Для расчета ранее валидированного показателя индивидуальной жизнеспособности использовались данные о 21 783 участниках 1-й (2011) и 2-й (2013) волн Продольного исследования здоровья и выхода на пенсию в Китае, которые также участвовали в опросе 2014 года CHARLS Life History Survey. Было рассмотрено 11 факторов в раннем периоде жизни, и была исследована их прямая связь с индивидуальной жизнеспособностью участников в более позднем периоде жизни, а также их косвенная связь через четыре текущих социально-экономических фактора. Для исследования вклада каждой детерминанты в неравенство в отношении индивидуальной жизнеспособности использовалась многомерная линейная регрессия и декомпозиция индекса концентрации.

**Результаты:**

У участников, находившихся в благоприятной окружающей среде в раннем возрасте (то есть образование родителей, здоровье в детстве и окружение в районе проживания), показатель индивидуальной жизнеспособности в последующей жизни был значительно выше. Например, у участников, чьи отцы образованны, показатель индивидуальной жизнеспособности был на 0,040 (95%-й ДИ: от 0,020 до 0,051) выше по сравнению с участниками, чьи отцы были неграмотными. Это неравенство оказалось более значительным для когнитивных, сенсорных и психологических способностей, чем для двигательной и жизненной активности. В целом факторы раннего периода жизни напрямую объясняют 13,92% (95%-й ДИ: от 12,07 до 15,77) неравенства в отношении индивидуальной жизнеспособности и еще 28,57% (95%-й ДИ: от 28,19 до 28,95) этого неравенства через их влияние на текущее социально-экономическое неравенство.

**Вывод:**

Неблагоприятные факторы в раннем периоде жизни снижают состояние здоровья после выхода на пенсию в Китае, особенно когнитивные, сенсорные и психологические способности, и эти эффекты усугубляются кумулятивным социально-экономическим неравенством на протяжении всей жизни человека.

## Introduction

A growing number of researchers consider traditional disease-based conceptualizations of health or health metrics based on single domains to be inadequate proxies for the health of older adults.^1^ Recognizing the importance of healthy ageing, the World Health Organization (WHO) used a capabilities approach to frame healthy ageing in the broadest possible sense in its 2015 *World report on ageing and health*.^2^ In the framework presented in the report, healthy ageing is experienced when the functional ability to be and do the things a person values is developed and maintained across a person’s life course. Functional ability has many dimensions, including being able to meet one’s daily basic needs; to learn, grow and make critical decisions; to be actively mobile; to build and maintain meaningful relationships; and to contribute to society.^3^ This ability is determined by a person’s intrinsic capacity, the environment they live in, and the interaction between the individual and their environment.^1^^,^^2^

Intrinsic capacity comprises all of the individual-level capacities that a person can draw on. Developing and maintaining these capacities across a person’s life course is critical to healthy ageing.^4^ Building on gerontological and disability theory,^5^ intrinsic capacity reflects the biological capacities of an individual rather than its impairments or deficits. This strengths-based approach is distinct from, but complementary to, concepts such as frailty and resilience.^6^ Clinicians and researchers have increasingly adopted intrinsic capacity to measure healthy ageing,^7^^,^^8^ and the construct has been successfully validated and empirically examined in longitudinal analyses of English^9^ and Chinese cohorts.^10^

Intrinsic capacity differs in several ways from other ageing concepts. First, intrinsic capacity potentially provides a continuous, fine-grained and comprehensive measure of overall health that can be assessed across much of the life course, rather than focusing on late-life impairments or impairment of specific body functions.^11^ Second, it adopts a functioning-oriented approach rather than a traditional disease-centred approach to framing health states.^12^ Third, apart from other comprehensive measures of overall health,^13^^,^^14^ it aims to distinguish individual attributes of functioning from the influence of the environment a person lives in. These characteristics facilitate cross-time, cross-country and cross-culture comparisons of healthy ageing and enable the investigation of the contextual attributes that may either affect intrinsic capacity or, in conjunction with intrinsic capacity, contribute to an individual’s functional ability. Researchers have suggested that regular monitoring of intrinsic capacity could provide an early warning for reduced functioning or adverse outcomes, and inform preventive interventions.^11^^,^^15^

In functional phenotype research, researchers are increasingly recognizing life-course analyses, which conceptualize an accumulation of risk as the incremental burden of different exposures with age, highlighting the importance of timing and cumulative exposure.^16^ These exposures may play a role in determining health through overt physiological change (e.g. weight loss due to famine) or through internal changes (e.g. regulation of the growth hormone insulin in relation to dietary exposure). Intrinsic capacity is likely to be shaped by many life-course factors, including underlying age-related biological changes, health-related behaviours and the presence of disease.^3^ Intrinsic capacity can also be influenced by socioeconomic and physical determinants that occur at different life stages. Moreover, disadvantages in nutrition, health, wealth, education, employment and income that arise early in life can reinforce each other and accumulate over the life course.^17^ The large heterogeneity in intrinsic capacity observed in older adults is thus likely to strongly reflect the accumulated impact of these determinants,^18^^,^^19^ inextricably linking poorer healthy ageing trajectories to early-life inequalities. However, the cumulative contribution of health and socioeconomic inequalities over time to intrinsic capacity levels in late life has not been well studied. To fill the gap, this study aims to longitudinally examine the relationships between life-course factors and intrinsic capacity in a representative sample of China’s older adult population.

## Methods

### Data and study participants

We used publicly available de-identified data from the China Health and Retirement Longitudinal Study (CHARLS) to measure the intrinsic capacity construct and to examine life-course inequalities within it.^20^ The study aims to collect high-quality data from a nationally representative sample of older Chinese adults aged 45 years or older.^21^ The first CHARLS cohort was recruited in 2011, with follow-up surveys conducted every two years. The 2011 nationally representative baseline sampling frame included 150 counties or districts and 450 villages or urban communities across China. All living respondents from the first two waves (2011 and 2013) were invited to participate in the 2014 CHARLS Life History Survey, which included a series of questions about health and health-care history, residential history, education history and important childhood events. We included all individuals who participated in the 2011 and 2013 waves and the 2014 CHARLS Life History Survey. 

### Intrinsic capacity

We have previously validated and described prognostic value of intrinsic capacity in this cohort.^10^ In summary, we calculated the intrinsic capacity score using structural equation models and confirmatory factor analysis of all objective characteristics that might be associated with the intrinsic capacity construct, including forced expiratory volume; chair-stand test; grip strength; balance; walking speed; haemoglobin; episodic memory test; affective, hearing and vision impairments; intact mental status; and sleep quantity and quality. The final construct includes an overarching domain, that is, intrinsic capacity, and five subdomains: locomotion; cognitive capacity; vitality; sensory capacity; and psychological capacity, with a mean standardized score of 0 and a standard deviation of 1. Higher scores indicate a higher capacity to do the things that individuals have reason to value.

### Life-course factors

We included 11 early-life factors that happened in critical periods of individuals’ lives (generally before the age of 17 years) based on the existing literature:^22^^–^^24^ (i) parental education; (ii) self-reported health status during childhood; (iii) access to health-care services; (iv) healthy behaviour; (v) nutritional status; (vi) family economic status during childhood; (vii) neighbourhood quality; (viii) friendships; (ix) parental mental health; (x) domestic violence; and (xi) receiving mentorship and support. Early-life factors can directly affect late-life intrinsic capacity, indirectly influence intrinsic capacity via their influence on the subsequent accumulation of socioeconomic inequalities, or both. We used four measures to account for current socioeconomic factors: (i) current family economic status; (ii) educational achievement; (iii) urban residence; and (iv) urban *hukou* registration. *Hukou* is a registration in the Chinese household registration system. More details on the definitions and measurements of these factors are available in the online repository.^25^

### Statistical analysis

First, we examined the unadjusted association of intrinsic capacity with each life-course factor of interest in a bivariate analysis.^26^ We then examined the correlation between early-life factors and current socioeconomic status using a Spearman correlation matrix, since early-life factors may affect late-life intrinsic capacity via accumulative health and socioeconomic inequalities.

Second, we used multivariable linear regressions to examine how life-course factors affect late-life intrinsic capacity, adjusting for current socioeconomic, demographic and lifestyle factors (online repository).^25^ We expressed associations as marginal effects measured at the mean of each covariate. As biases may arise in the estimated relationship between intrinsic capacity and factors of interest when the outcome variable and error term are correlated, our primary econometric strategy was to exploit the comprehensive information in the survey data to directly account for as many potential confounding factors as possible.^27^

Third, we used the concentration index to quantify inequalities in late-life intrinsic capacity.^28^ As a rank-dependent method, the concentration index was calculated based on family economic status.^29^^,^^30^ Moreover, we decomposed the concentration index of intrinsic capacity to quantify each determinant’s specific contribution and summarized them by categories (further details available in the online repository).^25^ Additionally, by excluding current socioeconomic factors from our models, we performed mediation analysis to identify the cumulative contribution of early-life factors to intrinsic capacity inequalities through health and socioeconomic inequalities over a person’s life course.

Finally, we performed sensitivity analyses to ensure the reliability of our findings (online repository).^25^ First, since a moderate proportion of participants was excluded, we used an incidental truncation model by introducing the inverse Mills ratio to adjust for potential selection bias.^31^ Second, we used multiple imputations with chained equations to impute missing values,^32^ assuming observations were missing at random. We combined estimates across 20 imputed data sets based on Rubin’s rules.^33^ Third, to ensure the relationships were consistent in both older and younger participants, we used a chronologically pre-old group (younger than 60 years) and an older group (aged 60 years or older) to perform heterogeneity analysis. Fourth, to assess whether recall bias among participants affected our results, we performed heterogeneity analysis by splitting the sample into a low-cognition and a high-cognition subgroup. Fifth, we excluded duplicated participants by prioritizing data in 2013, and used family income per capita rather than family consumption. Sixth, we replicated our analysis using the latest wave of CHARLS in 2018. We did not include this wave in our main analysis since, by design, several key intrinsic capacity measures were not collected, and participants added in the 2015 and 2018 waves did not complete the 2014 CHARLS Life History Survey. Seventh, we examined the validity of early-life factors using data from the 2011 CHARLS community questionnaire, which was completed by a respondent from a village committee office or a community committee.^34^

We reported two-sided *P*-values (unadjusted for multiple comparisons) and 95% confidence intervals (CIs). We considered a *P*-value < 0.05 to be statistically significant, and we used the Holm-Bonferroni method to adjust for multiple comparisons. All analyses were performed in Stata version 16.0 (Stata Corp LLP, College Station, United States of America).

### Ethical considerations

The Biomedical Ethics Review Committee of Peking University approved the CHARLS study (IRB00001052–11015), and all the data is publicly available. Furthermore, the current study received approval from the University of New South Wales (UNSW) Ethics Committee (HC210472).

## Results

After excluding 117 individuals who did not answer the intrinsic capacity questions, and 7651 and 941 who had missing information on life history or socioeconomic factors, respectively, we included 21 783 survey participants. The mean age of the participants was 59.07 years (standard deviation, SD: 9.16) and 10 600 participants (48.66%) were male. The sample included 9997 participants in the older age group (range: 60–109 years; mean: 67.15; SD: 6.01) and 11 786 participants in the younger age group (range:19–59 years; mean: 52.22; SD: 4.71). Table 1 shows the characteristics of the included participants. The 8709 excluded participants were more likely to have a lower family economic status, a lower education level and not having urban *hukou*. Also, the excluded participants were more likely to be older, single, female, more likely to consume tobacco and alcohol, and more likely to have chronic diseases (online repository).^25^

**Table 1:** Characteristics of participants included in the study on intrinsic capacity and healthy ageing, China, 2011–2013

Characteristic	No. (%) (*n* = 21 783)
**Gender^a^**	
Female	11 183 (51.3)
Male	10 600 (48.7)
**Age group, years**	
< 45	385 (1.8)
45–60	11 401 (52.3)
60–70	6 951 (31.9)
70–80	2 674 (12.3)
≥ 80	372 (1.7)
**Marital status**	
Married	18 310 (84.1)
Not married	3 473 (15.9)
**Residence**	
Rural	15 455 (70.9)
Urban	6 328 (29.1)
**Education level**	
Illiterate	5 385 (24.7)
Primary	8 911 (40.9)
Middle school	4 773 (21.9)
High school	2 586 (11.9)
College and above	128 (0.6)
**Urban *hukou*^b^**	
Agriculture	17 194 (78.9)
Non-agriculture	4 589 (21.1)
**Family economic status, wealth quintile**	
1: poorest	4 387 (20.1)
2: poor	4 539 (20.8)
3: average	4 410 (20.2)
4: richer	4 309 (19.8)
5: richest	4 138 (19.0)
**Tobacco consumption**	
No	13 875 (63.7)
Yes	7 908 (36.3)
**Alcohol consumption**	
No	14 105 (64.8)
Yes	7 678 (35.2)
**Presence of chronic diseases**	
No	7 285 (33.4)
Yes	14 498 (66.6)

^a^ The interviewer recorded respondent’s gender as either female or male.

^b^ *Hukou* is an individual registered in the Chinese household registration system.

Note: Inconsistencies arise in some values due to rounding.

**Fig. 1:**
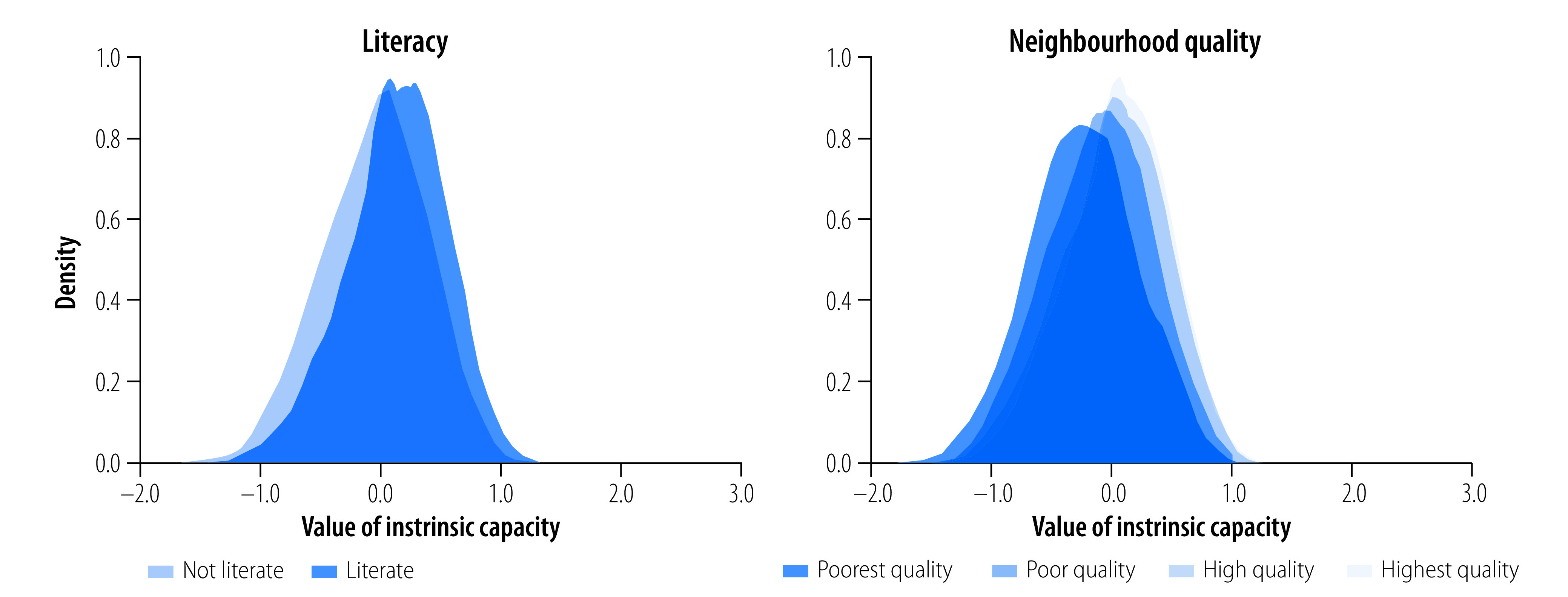
Distribution of intrinsic capacity and the association of life-course factors, China, 2011–2013

Notes: We included 21 783 participants from the CHARLS study, waves 1 and 2. The plot shows Kernel density distribution of intrinsic capacity. Neighbourhood quality was assessed by four questions asking participants whether the neighbourhood where they lived as a child was safe, willing to help, close-knit, and clean and attractive. Each measure was dichotomised as low (not very or not at all) and high (somewhat or very) leading to a sum measure of adversity ranging from 0 (lowest) to 4 (highest).

**Fig. 2:**
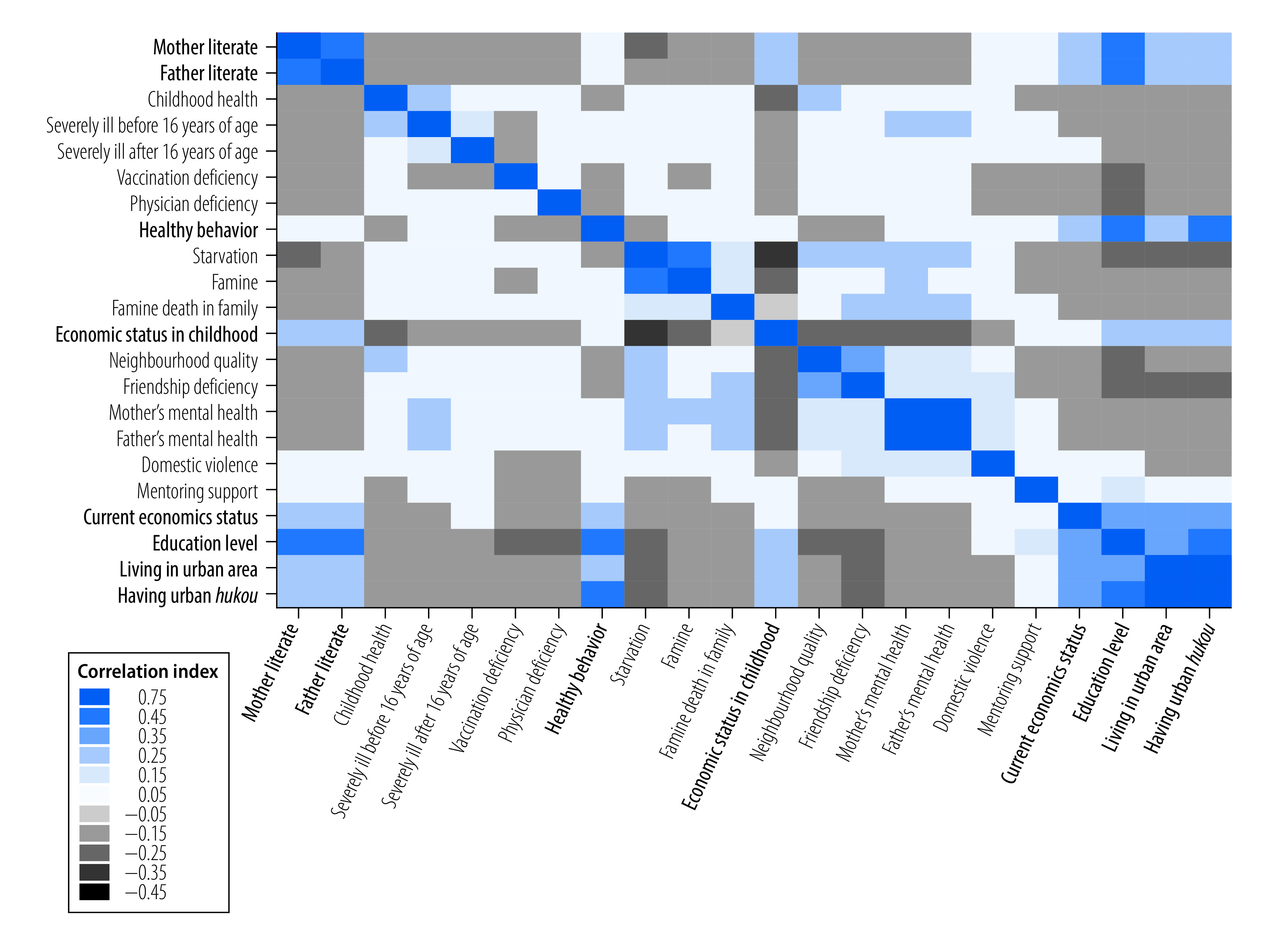
Pairwise Spearman correlation matrix between life-course factors, China, 2011–2013

Notes: We included 21 783 participants from the CHARLS study, waves 1 and 2. Variables in bold denote positive measures with higher socioeconomic position or childhood advantages. *Hukou* is an individual registered in the Chinese household registration system. Definitions of the variables are available in the online repository.^25^

We first plotted Kernel density distributions to explore the association between intrinsic capacity and early-life factors (Fig. 1). The plots showed that participants with lower parental education and worse neighbourhood environment had lower intrinsic capacity scores than more advantaged participants. Other early-life factors and the subdomains of intrinsic capacity showed similar patterns (*P*-value: < 0.001; online repository).^25^ The multivariate analysis using all early-life factors also supported these results (online repository).^25^ Intrinsic capacity was also significantly associated with current socioeconomic, demographic and lifestyle factors (*P*-value: < 0.001; online repository).^25^ The Spearman correlation matrix showed reasonably moderate and strong correlations for factors across life-course stages (Fig. 2).

**Fig. 3:**
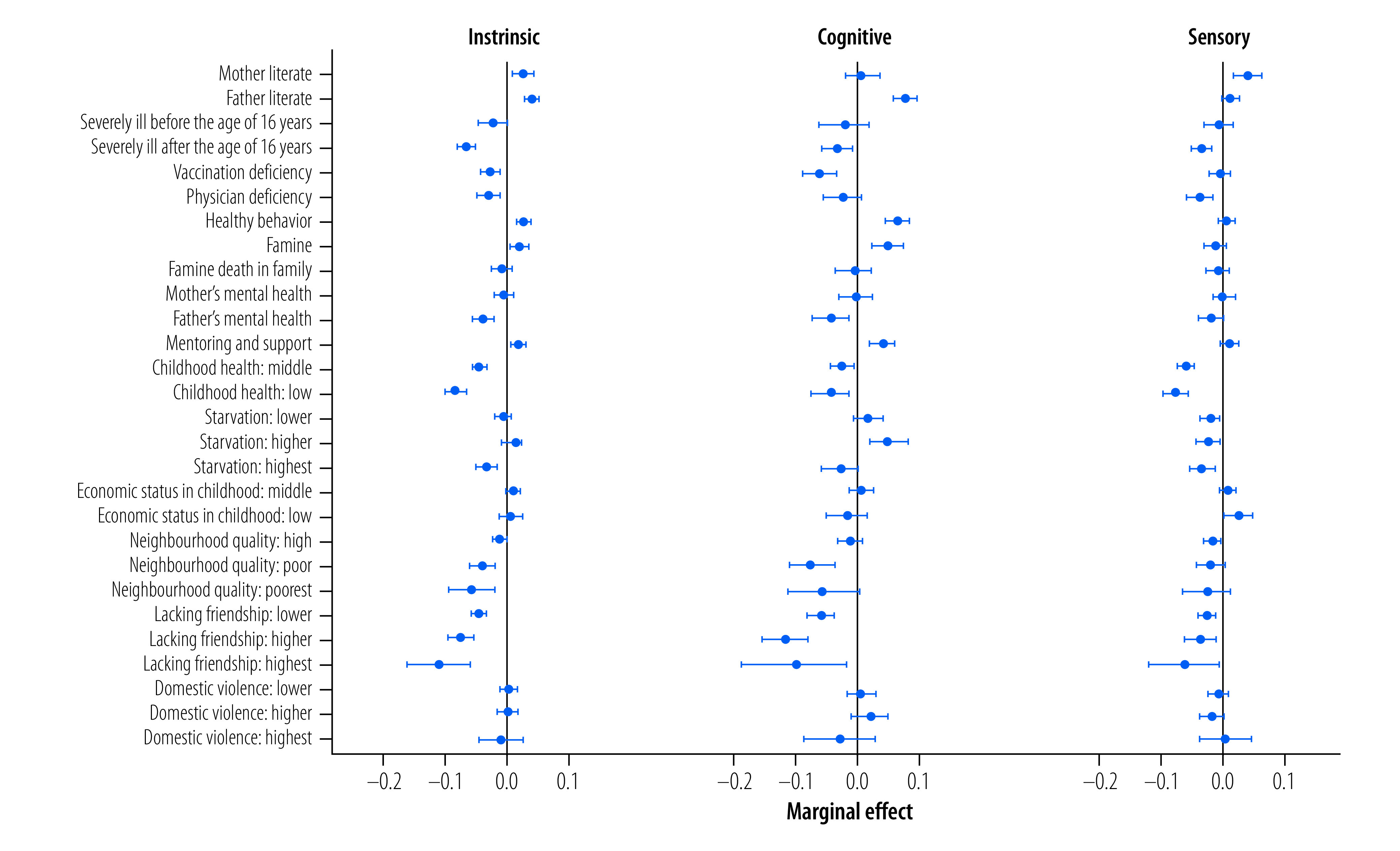
Linking early-life factors with late-life intrinsic, cognitive and sensory capacities, China, 2011–2013

We further investigated the association between intrinsic capacity and life-course factors. In the regression analysis, early-life factors were significantly associated with intrinsic capacity later in life, even after eliminating the effect of current socioeconomic, demographic and lifestyle factors (Fig. 3). For example, people with a literate father recorded a 0.040 (95% CI: 0.020 to 0.051) higher intrinsic capacity score than those with an illiterate father (Table 2; available at: https://www.who.int/publications/journals/bulletin/). People living in neighbourhoods with worse environment and who were lacking friends during childhood recorded significantly lower intrinsic capacity scores. Moreover, higher current socioeconomic positions were significantly associated with higher intrinsic capacity scores for participants with a higher family economic status, living in urban areas, having an urban *hukou* or having attained higher education attainments (Table 2).

CI: confidence interval.

Notes: We included 21 783 participants from the CHARLS study, waves 1 and 2. Multivariate linear regressions were performed by adjusting for current socioeconomic factors, lifestyle factors and demographic factors. Intrinsic capacity represents the overall level of health and capacities to be and do things the participant has reasons to value cognitive capacity includes memory, intelligence and problem-solving; sensory capacity includes vision and hearing; psychological capacity involves mood and emotional resilience. Table 2 presents all the marginal effects and their associated 95% CIs.

**Table 2:** Association between life-course factors and intrinsic capacity and its subdomains, China, 2011–2013

Variable	Marginal effect (95% CI)					
	Intrinsic capacity	Locomotion	Cognitive	Vitality	Sensory	Psychological
**Gender^a^**						
Female	Reference	Reference	Reference	Reference	Reference	Reference
Male	0.236 (0.222 to 0.250)	0.031 (−0.022 to 0.083)	0.119 (0.095 to 0.144)	0.424 (0.407 to 0.440)	0.079 (0.063 to 0.096)	0.427 (0.367 to 0.487)
**Age group, years**						
< 45	Reference	Reference	Reference	Reference	Reference	Reference
45–60	−0.124 (−0.159 to −0.088)	−0.089 (−0.148 to −0.029)	−0.095 (−0.157 to −0.033)	−0.049 (−0.084 to −0.014)	−0.187 (−0.233 to −0.140)	−0.109 (−0.249 to 0.032)
60–70	−0.222 (−0.259 to −0.185)	−0.216 (−0.284 to −0.148)	−0.142 (−0.206 to −0.078)	−0.190 (−0.227 to −0.154)	−0.255 (−0.303 to −0.207)	−0.105 (−0.251 to 0.040)
70–80	−0.346 (−0.386 to −0.307)	−0.513 (−0.606 to −0.419)	−0.253 (−0.321 to −0.185)	−0.347 (−0.385 to −0.308)	−0.303 (−0.353 to −0.252)	−0.126 (−0.281 to 0.029)
≥ 80	−0.525 (−0.585 to −0.466)	−1.411 (−1.714 to −1.108)	−0.424 (−0.516 to −0.332)	−0.458 (−0.520 to −0.396)	−0.351 (−0.418 to −0.285)	−0.236 (−0.460 to −0.012)
**Marital status**						
Married	Reference	Reference	Reference	Reference	Reference	Reference
Single	−0.056 (−0.070 to −0.042)	−0.045 (−0.095 to 0.005)	−0.073 (−0.097 to −0.048)	−0.044 (−0.058 to −0.030)	−0.003 (−0.019 to 0.013)	−0.165 (−0.223 to −0.108)
**Education level**						
No education	Reference	Reference	Reference	Reference	Reference	Reference
Primary school	0.193 (0.178 to 0.208)	0.151 (0.101 to 0.201)	0.563 (0.537 to 0.590)	0.025 (0.011 to 0.040)	−0.004 (−0.020 to 0.013)	0.025 (−0.036 to 0.085)
Middle school	0.302 (0.285 to 0.320)	0.168 (0.111 to 0.226)	0.815 (0.784 to 0.846)	0.072 (0.053 to 0.091)	0.027 (0.006 to 0.047)	0.030 (−0.044 to 0.103)
High school	0.361 (0.339 to 0.382)	0.182 (0.113 to 0.252)	0.897 (0.860 to 0.933)	0.086 (0.060 to 0.111)	0.065 (0.039 to 0.092)	0.119 (0.028 to 0.209)
College and above	0.412 (0.353 to 0.472)	0.183 (−0.028 to 0.394)	0.952 (0.874 to 1.030)	0.038 (−0.036 to 0.111)	0.097 (0.011 to 0.182)	0.242 (0.006 to 0.478)
**Residence**						
Rural	Reference	Reference	Reference	Reference	Reference	Reference
Urban	0.048 (0.034 to 0.062)	0.001 (−0.047 to 0.049)	0.093 (0.069 to 0.117)	0.019 (0.002 to 0.036)	0.027 (0.010 to 0.045)	−0.062 (−0.119 to −0.004)
**Urban *hukou*^b^**						
No	Reference	Reference	Reference	Reference	Reference	Reference
Yes	0.062 (0.046 to 0.079)	0.006 (−0.048 to 0.061)	0.114 (0.087 to 0.142)	0.012 (−0.009 to 0.032)	0.032 (0.012 to 0.052)	0.048 (−0.020 to 0.116)
**Family economic status**						
Poorest	Reference	Reference	Reference	Reference	Reference	Reference
Poorer	0.032 (0.017 to 0.047)	0.060 (0.006 to 0.113)	0.055 (0.028 to 0.081)	0.032 (0.016 to 0.049)	0.013 (−0.005 to 0.030)	−0.035 (−0.095 to 0.025)
Average	0.044 (0.029 to 0.059)	0.033 (−0.021 to 0.086)	0.064 (0.037 to 0.091)	0.037 (0.020 to 0.053)	0.032 (0.014 to 0.050)	−0.006 (−0.067 to 0.055)
Richer	0.048 (0.032 to 0.063)	0.007 (−0.049 to 0.063)	0.087 (0.059 to 0.114)	0.037 (0.020 to 0.054)	0.032 (0.014 to 0.050)	−0.017 (−0.080 to 0.045)
Richest	0.066 (0.049 to 0.082)	−0.003 (−0.065 to 0.058)	0.124 (0.095 to 0.152)	0.037 (0.019 to 0.055)	0.053 (0.034 to 0.073)	−0.030 (−0.095 to 0.036)
**Literacy**						
Mother illiterate	Reference	Reference	Reference	Reference	Reference	Reference
Mother literate	0.027 (0.009 to 0.044)	0.040 (−0.005 to 0.084)	0.007 (−0.021 to 0.035)	0.020 (−0.002 to 0.042)	0.040 (0.019 to 0.061)	−0.010 (−0.083 to 0.063)
Father illiterate	Reference	Reference	Reference	Reference	Reference	Reference
Father literate	0.040 (0.029 to 0.051)	0.035 (0.001 to 0.069)	0.077 (0.057 to 0.096)	0.024 (0.011 to 0.036)	0.012 (−0.001 to 0.025)	0.043 (−0.003 to 0.089)
**Childhood health**						
General						
High	Reference	Reference	Reference	Reference	Reference	Reference
Average	−0.043 (−0.054 to −0.032)	−0.003 (−0.038 to 0.032)	−0.026 (−0.045 to −0.007)	−0.031 (−0.044 to −0.019)	−0.061 (−0.074 to −0.047)	−0.059 (−0.106 to −0.012)
Low	−0.082 (−0.099 to −0.065)	−0.032 (−0.090 to 0.027)	−0.046 (−0.076 to −0.016)	−0.074 (−0.093 to −0.055)	−0.077 (−0.098 to −0.057)	−0.218 (−0.291 to −0.145)
Severely ill before the age of 16 years						
No	Reference	Reference	Reference	Reference	Reference	Reference
Yes	−0.022 (−0.046 to 0.001)	−0.031 (−0.106 to 0.043)	−0.021 (−0.062 to 0.019)	−0.006 (−0.030 to 0.018)	−0.008 (−0.033 to 0.017)	−0.061 (−0.158 to 0.035)
Severely ill after the age of 16 years						
No	Reference	Reference	Reference	Reference	Reference	Reference
Yes	−0.064 (−0.079 to −0.050)	−0.201 (−0.258 to −0.144)	−0.034 (−0.059 to −0.010)	−0.046 (−0.062 to −0.030)	−0.035 (−0.052 to −0.019)	−0.199 (−0.258 to −0.139)
**Access to health-care services**						
Vaccination deficiency^c^						
No	Reference	Reference	Reference	Reference	Reference	Reference
Yes	−0.027 (−0.042 to −0.012)	0.014 (−0.037 to 0.065)	−0.063 (−0.090 to −0.035)	−0.019 (−0.035 to −0.004)	−0.006 (−0.023 to 0.011)	−0.008 (−0.072 to 0.057)
Physician deficiency^d^						
No	Reference	Reference	Reference	Reference	Reference	Reference
Yes	−0.030 (−0.048 to −0.012)	0.026 (−0.033 to 0.084)	−0.026 (−0.057 to 0.005)	−0.005 (−0.024 to 0.014)	−0.038 (−0.058 to −0.018)	−0.069 (−0.143 to 0.004)
**Healthy behaviour**						
No	Reference	Reference	Reference	Reference	Reference	Reference
Yes	0.027 (0.016 to 0.039)	0.018 (−0.018 to 0.053)	0.064 (0.044 to 0.084)	0.010 (−0.003 to 0.023)	0.006 (−0.008 to 0.019)	0.041 (−0.005 to 0.088)
**Starvation**						
None	Reference	Reference	Reference	Reference	Reference	Reference
Low	−0.006 (−0.020 to 0.007)	0.031 (−0.014 to 0.076)	0.016 (−0.008 to 0.040)	0.000 (−0.016 to 0.016)	−0.021 (−0.038 to −0.005)	−0.088 (−0.145 to −0.030)
Moderate	0.007 (−0.009 to 0.024)	0.038 (−0.015 to 0.091)	0.049 (0.019 to 0.078)	0.009 (−0.010 to 0.028)	−0.024 (−0.044 to −0.004)	−0.042 (−0.111 to 0.028)
Severe	−0.033 (−0.050 to −0.016)	0.019 (−0.037 to 0.076)	−0.030 (−0.059 to −0.000)	−0.010 (−0.028 to 0.008)	−0.035 (−0.054 to −0.015)	−0.153 (−0.224 to −0.082)
**Famine**						
No	Reference	Reference	Reference	Reference	Reference	Reference
Yes	0.020 (0.006 to 0.035)	0.036 (−0.014 to 0.087)	0.047 (0.022 to 0.072)	0.031 (0.016 to 0.047)	−0.012 (−0.030 to 0.006)	0.006 (−0.055 to 0.068)
**Famine death in family**						
No	Reference	Reference	Reference	Reference	Reference	Reference
Yes	−0.008 (−0.025 to 0.009)	0.047 (−0.006 to 0.100)	−0.008 (−0.038 to 0.021)	0.013 (−0.005 to 0.032)	−0.009 (−0.028 to 0.010)	−0.094 (−0.169 to −0.020)
**Economic status in childhood**						
High	Reference	Reference	Reference	Reference	Reference	Reference
Average	0.010 (−0.001 to 0.022)	0.012 (−0.025 to 0.049)	0.006 (−0.014 to 0.026)	0.005 (−0.008 to 0.018)	0.008 (−0.005 to 0.021)	0.033 (−0.015 to 0.081)
Low	0.007 (−0.012 to 0.025)	0.003 (−0.057 to 0.064)	−0.019 (−0.051 to 0.014)	−0.017 (−0.039 to 0.005)	0.024 (0.001 to 0.047)	0.075 (−0.005 to 0.155)
**Neighbourhood quality**						
Highest	Reference	Reference	Reference	Reference	Reference	Reference
High	−0.012 (−0.023 to −0.000)	−0.020 (−0.056 to 0.017)	−0.013 (−0.032 to 0.007)	0.003 (−0.010 to 0.016)	−0.018 (−0.031 to −0.004)	−0.026 (−0.074 to 0.021)
Poor	−0.039 (−0.059 to −0.020)	0.004 (−0.059 to 0.067)	−0.075 (−0.111 to −0.038)	−0.021 (−0.043 to −0.000)	−0.020 (−0.043 to 0.003)	−0.024 (−0.108 to 0.060)
Poorest	−0.056 (−0.092 to −0.019)	−0.111 (−0.255 to 0.032)	−0.056 (−0.115 to 0.002)	−0.048 (−0.082 to −0.015)	−0.027 (−0.065 to 0.011)	−0.052 (−0.181 to 0.077)
**Friendship deficiency**						
Low	Reference	Reference	Reference	Reference	Reference	Reference
Lower	−0.045 (−0.057 to −0.033)	−0.057 (−0.096 to −0.017)	−0.061 (−0.082 to −0.040)	−0.024 (−0.038 to −0.011)	−0.027 (−0.040 to −0.013)	−0.049 (−0.099 to 0.001)
Higher	−0.073 (−0.094 to −0.053)	−0.016 (−0.087 to 0.056)	−0.118 (−0.156 to −0.081)	−0.027 (−0.050 to −0.003)	−0.037 (−0.061 to −0.013)	−0.151 (−0.240 to −0.062)
Highest	−0.109 (−0.159 to −0.059)	−0.031 (−0.186 to 0.123)	−0.105 (−0.190 to −0.019)	−0.028 (−0.072 to 0.017)	−0.064 (−0.121 to −0.007)	−0.321 (−0.511 to −0.131)
**Parental mental health**						
Mother						
Good	Reference	Reference	Reference	Reference	Reference	Reference
Poor	−0.004 (−0.020 to 0.011)	0.021 (−0.024 to 0.067)	−0.003 (−0.030 to 0.023)	0.018 (0.001 to 0.034)	0.001 (−0.017 to 0.019)	−0.107 (−0.173 to −0.041)
Father						
Good	Reference	Reference	Reference	Reference	Reference	Reference
Poor	−0.038 (−0.055 to −0.021)	−0.026 (−0.079 to 0.026)	−0.045 (−0.075 to −0.016)	−0.023 (−0.041 to −0.005)	−0.020 (−0.040 to −0.000)	−0.062 (−0.133 to 0.009)
**Domestic violence**						
Low	Reference	Reference	Reference	Reference	Reference	Reference
Lower	0.003 (−0.011 to 0.017)	−0.007 (−0.052 to 0.038)	0.005 (−0.018 to 0.029)	0.014 (−0.001 to 0.029)	−0.007 (−0.024 to 0.009)	−0.050 (−0.109 to 0.008)
Higher	0.001 (−0.016 to 0.018)	0.012 (−0.038 to 0.061)	0.018 (−0.010 to 0.047)	0.018 (−0.003 to 0.040)	−0.019 (−0.038 to 0.000)	−0.109 (−0.179 to −0.039)
Highest	−0.009 (−0.045 to 0.026)	0.016 (−0.091 to 0.123)	−0.029 (−0.086 to 0.028)	0.041 (0.009 to 0.074)	0.003 (−0.039 to 0.045)	−0.080 (−0.229 to 0.068)
**Mentoring and support**						
No	Reference	Reference	Reference	Reference	Reference	Reference
Yes	0.019 (0.007 to 0.031)	0.038 (0.004 to 0.073)	0.040 (0.019 to 0.060)	0.012 (−0.002 to 0.026)	0.011 (−0.003 to 0.026)	−0.014 (−0.064 to 0.035)
**Tobacco consumption**						
No	Reference	Reference	Reference	Reference	Reference	Reference
Yes	−0.027 (−0.040 to −0.014)	0.017 (−0.029 to 0.063)	−0.040 (−0.062 to −0.018)	−0.009 (−0.024 to 0.007)	−0.016 (−0.032 to −0.001)	−0.053 (−0.107 to 0.001)
**Alcohol consumption**						
No	Reference	Reference	Reference	Reference	Reference	Reference
Yes	0.025 (0.014 to 0.036)	0.053 (0.016 to 0.090)	0.006 (−0.013 to 0.026)	0.046 (0.033 to 0.059)	0.024 (0.010 to 0.038)	−0.006 (−0.054 to 0.041)
**Chronic disease**						
No	Reference	Reference	Reference	Reference	Reference	Reference
Yes	−0.085 (−0.096 to −0.074)	−0.097 (−0.127 to −0.066)	−0.022 (−0.041 to −0.003)	−0.034 (−0.046 to −0.022)	−0.108 (−0.121 to −0.094)	−0.326 (−0.371 to −0.281)

^a^ The interviewer recorded respondent’s gender as either female or male.

^b^ *Hukou* is an individual registered in the Chinese household registration system.

^c^ Vaccination deficiency was assessed by the question: before or when you were 15 years old, did you receive any vaccinations?

^d^ Physician deficiency was assessed by the question: have you always had a usual source of care, that is, a particular person or a place that you went to when you were sick or needed advice about your health?

Note: Further information on the measurement of variables is available in the online repository.^25^

**Fig. 4:**
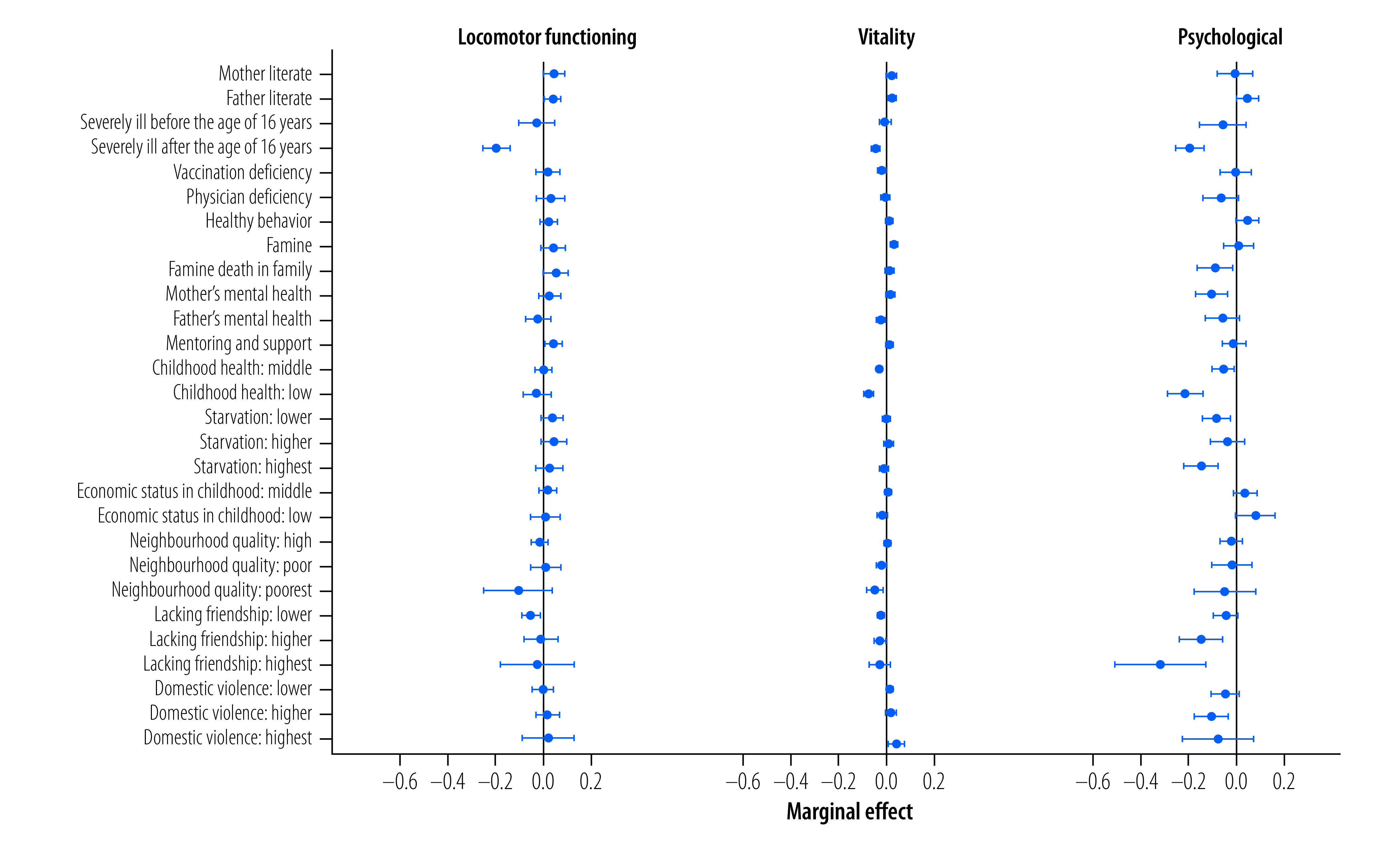
Linking early-life factors with late-life locomotion, vitality and psychological capacity, China, 2011–2013

The strongest associations of childhood disadvantages were with lower cognitive, sensory and psychological capacities, although we also identified negative effects for locomotion and vitality (Table 2, Fig. 3 and Fig. 4). Most estimated marginal effects for cognitive, sensory and psychological capacities were statistically significant. For example, persons with a literate father recorded a 0.077 (95% CI: 0.057 to 0.096) higher cognitive capacity score than those with an illiterate father (Table 2 and Fig. 3). However, the estimated marginal effects for vitality were small, if statistically significant, and most estimated marginal effects for locomotion were not statistically significant.

CI: confidence interval.

Notes: We included 21 783 participants from the CHARLS study, waves 1 and 2. Multivariate linear regressions were performed by adjusting for current socioeconomic factors, lifestyle factors and demographic factors. Locomotion includes balance, muscle strength and gait; vitality is defined as underlying a person’s resilience to challenges, vigour and stamina, to maintain homeostasis in the face of daily stressors; psychological capacity involves mood and emotional resilience. Table 2 presents all the marginal effects and their associated 95% CIs.

We quantified the extent of intrinsic capacity inequalities using the concentration index method. The concentration index was 0.024 (95% CI: 0.021 to 0.027) for intrinsic capacity (Table 3). The positive value indicates that the richer wealth groups had higher intrinsic capacity scores. Cognitive and sensory capacities had the highest concentration indices, indicating more inequalities in these capacities.

**Table 3:** Extent of inequalities in intrinsic capacity, China, 2011–2013

Capacity	Modified concentration index (95% CI)
Intrinsic	0.024 (0.021 to 0.027)
Locomotion	0.002 (−0.002 to 0.006)
Cognitive	0.043 (0.036 to 0.050)
Vitality	0.012 (0.011 to 0.013)
Sensory	0.032 (0.025 to 0.039)
Psychological	0.005 (−0.006 to 0.016)

CI: confidence interval.

Notes: We included 21 783 participants from the CHARLS study, waves 1 and 2. The concentration index ranges from −1 to 1, with positive values indicating that people with a higher intrinsic capacity are more concentrated among high-income groups and negative values indicating that people with a higher intrinsic capacity are more concentrated among low-income groups.

**Fig. 5:**
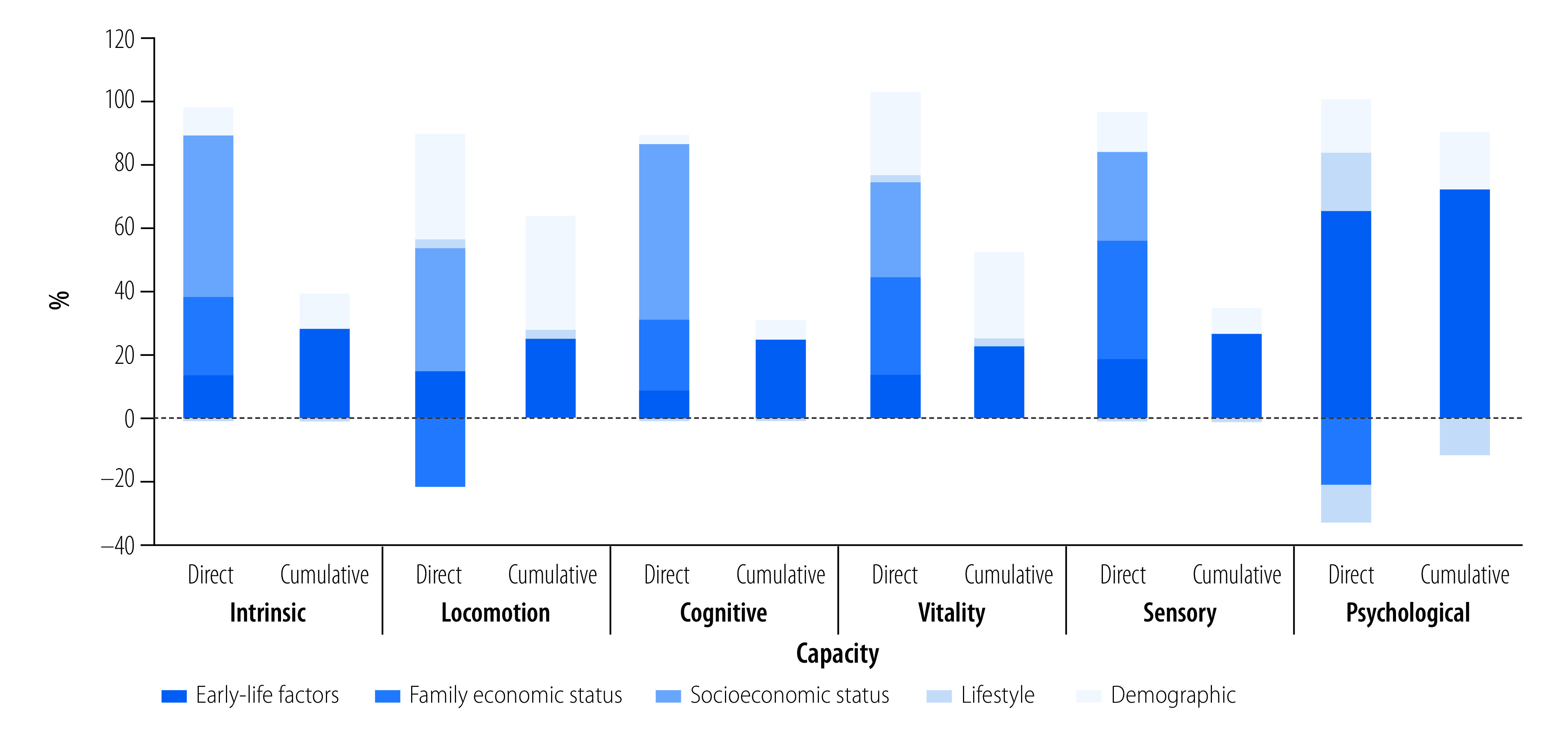
Decomposing the life-course inequalities in intrinsic capacity, China, 2011–2013

Notes: We included 21 783 participants from the CHARLS study, waves 1 and 2. The percentages show the contribution of each category factor to the overall intrinsic capacity inequalities by decomposing the modified concentration index. For bars showing direct contributions, all life course factors were included, and thus the graph shows the direct contribution of early-life factors to inequalities in intrinsic capacity. For bars showing cumulative contributions, current socioeconomic position was excluded from the model of mediation analysis, and thus the graph shows the cumulative contribution of early-life factors to inequalities in intrinsic capacity. Current socioeconomic position excludes the family economic status factor. Theoretically, the contribution of observable determinants to inequalities in intrinsic capacity can be more than 100% since the contribution of the error term is not included and can be negative. Further details are available in the online repository.^25^

We decomposed the concentration index into the contribution of each life-course factor to identify the comparative importance of these factors. Overall, early-life factors directly accounted for 13.92% (95% CI: 13.57 to 14.27) of intrinsic capacity inequalities (Fig. 5). The contribution of each early-life factor was similar across subdomains, with psychological capacity as the only exception (online repository).^25^ The decomposition for locomotion and psychological capacity was less precise, since the two subdomains only had small absolute values of the concentration index. In our mediation analysis, the marginal effect of early-life factors on intrinsic capacity generally became larger (online repository),^25^ leading early-life factors to account for 28.57% (95% CI: 28.19 to 28.95) of intrinsic capacity inequalities (Fig. 5). The details about each determinant’s contribution are reported in the online repository.^25^

We performed six theory-based sensitivity analyses. None of these substantially changed our results, suggesting that our results are robust and reliable (online repository).^25^

## Discussion

Using a nationally representative sample of the Chinese population, we find that unfavourable early-life factors were strongly linked to lower late-life intrinsic capacity scores. Approximately 14% of late-life intrinsic capacity inequalities could be explained by the direct effect of early-life factors, and a further 29% of intrinsic capacity inequalities through early-life factors’ influence on current socioeconomic inequalities. Unfavourable early-life factors were more likely to be associated with lower cognitive, sensory and psychological capacities and vitality than inequalities in locomotion.

Previous research has linked early-life factors to late-life health outcomes, including frailty, depression and biological ageing.^24^^,^^35^^,^^36^ However, this study represents a comprehensive life-course inequalities analysis of intrinsic capacity, a continuous and holistic construct of human health that is being increasingly applied in research and clinical practice.

The association of early-life factors with cognitive capacity are particularly interesting. There is growing evidence that, at least in western Europe, cognitive capacity in older adults is higher than in previous generations of the same age,^37^ although the underlying mechanisms remain unclear. Early brain development,^38^ such as child brain structural alterations of the cerebral cortex^39^ and the volume of the hippocampus,^40^ can often determine cognitive capacity in older age. The associations we found between many early-life factors (i.e. parental education and childhood health) and cognitive inequalities in late life can support this cohort difference.

The association of events early in life with vitality is consistent, with the suggestion that ambient stress caused by negative exposures results in biological ageing throughout a person’s life course.^41^ We have previously hypothesized that vitality is a marker of underlying biological changes associated with ageing and of an individual’s physiologic resilience.^10^

The main early-life factors associated with poor sensory capacity in late life concerned starvation and illness which may physiologically have affected the development of hearing and vision.

Our measure of psychological capacity was dominated by a depression score,^42^ which itself is heavily influenced by current life events and genetic predisposition.^43^ However, psychological capacity was partly dominated by early-life factors, although the marginal effects were seldom significant, suggesting an influence of childhood development on the capacity to manage stressors.

The smaller negative association of early-life adversities with locomotion are somewhat surprising. Locomotion is influenced by many factors, having modest associations with childhood socioeconomic position.^44^ Moreover, the dysregulation derived from stressors in childhood could be masked by the growth of organs since locomotion generally covers skeletal muscle physiology, cardiovascular system, energy homeostasis, osteoarticular and neurophysiology.^45^ While diminished locomotor capacity was also associated with adverse early exposures, the impact was less than for cognitive capacity.

Overall, our findings are consistent with extensive research across countries pointing to the important influence of socioeconomic determinants across the life course on different health inequalities.^46^ However, the outcomes considered by these studies have often been limited to mortality or specific conditions.^46^ Where functioning has been considered more broadly as an ageing-related outcome, it is often in relation to severe losses of functioning identified by a diagnosis of frailty or the inability to undertake basic activities without assistance.^24^^,^^47^ Most of these analyses did not study the contribution of life-course inequalities to health. In contrast, the intrinsic capacity construct allows us to explore the impact of life-course factors on the overall functioning of older adults, and to understand the different domains of functioning that are most affected.^24^^,^^35^^,^^48^

Decomposing intrinsic capacity inequalities into life-course factors may help identify key opportunities for proactive interventions to preserve intrinsic capacity in older age. Our findings emphasize the importance of equitable early-life opportunities on people’s subsequent capacity to make choices, contribute to society and receive support when needed.^3^ Almost all the determinants of health disparities can be reshaped by public policies across an individual’s life course. Our findings emphasize the importance of early-life events, which are largely experienced without choice soon after birth. This fact is critical for the individual and the society, since outcome inequalities are considered unfair if they are rooted in factors that are beyond individual control.^49^

Our analysis has many strengths. We used a representative sample of the world’s largest ageing population, the wealth of outcome data enabled us to explore health inequalities through a broad continuous measure of functioning, and the associated life events survey allowed us to distinguish between the impact of early- and late-life events. Moreover, the approach to measuring intrinsic capacity is consistent with a similar analysis of a large English cohort, suggesting the approach is robust across different cultural settings.^9^ However, the study has several limitations. First, we used individual recall data for early-life factors with potential recall bias, although our sensitivity analysis suggests that cognitive decline does not seem to significantly affect our findings. Second, this study could not distinguish whether intrinsic capacity variations were caused by higher or lower peaks of intrinsic capacity or the rate of declining speeds of such capacity. Accordingly, we will further examine the longitudinal nature of intrinsic capacity when new data becomes available. Third, potential cohort effects may limit the generalization of our findings, and this effect should be further explored in an analysis regarding the trajectories of intrinsic capacity. Fourth, genetic analysis was not included in the study; instead, we included a comprehensive set of variables to try to counteract individual heterogeneity. Finally, the study design precludes the interpretation of our results as causal relationships.

Despite these limitations, the results highlight the important and cumulative impact early-life advantages and disadvantages have on people’s experience of health in older age. Our findings also provide support for the Chinese government’s latest initiatives, such as common prosperity and rural revitalization, designed to reduce rural–urban disparities and wealth inequalities.
